# A need for exhaustive and standardized characterization of ion channels activity. The case of K_V_11.1

**DOI:** 10.3389/fphys.2023.1132533

**Published:** 2023-02-13

**Authors:** Malak Alameh, Barbara Ribeiro Oliveira-Mendes, Florence Kyndt, Jordan Rivron, Isabelle Denjoy, Florian Lesage, Jean-Jacques Schott, Michel De Waard, Gildas Loussouarn

**Affiliations:** ^1^ CNRS, INSERM, l’institut du thorax, Nantes Université, CHU Nantes, Nantes, France; ^2^ Labex ICST, INSERM, CNRS, Institut de Pharmacologie Moléculaire et Cellulaire, Université Côte d’Azur, Valbonne, France; ^3^ Service de Cardiologie et CNMR Maladies Cardiaques Héréditaires Rares, Hôpital Bichat, Paris, France

**Keywords:** *KCNH2* gene, variants, electrophysiology, long QT syndrome, phenotyping

## Abstract

hERG, the pore-forming subunit of the rapid component of the delayed rectifier K^+^ current, plays a key role in ventricular repolarization. Mutations in the *KCNH2* gene encoding hERG are associated with several cardiac rhythmic disorders, mainly the Long QT syndrome (LQTS) characterized by prolonged ventricular repolarization, leading to ventricular tachyarrhythmias, sometimes progressing to ventricular fibrillation and sudden death. Over the past few years, the emergence of next-generation sequencing has revealed an increasing number of genetic variants including *KCNH2* variants. However, the potential pathogenicity of the majority of the variants remains unknown, thus classifying them as variants of uncertain significance or VUS. With diseases such as LQTS being associated with sudden death, identifying patients at risk by determining the variant pathogenicity, is crucial. The purpose of this review is to describe, on the basis of an exhaustive examination of the 1322 missense variants, the nature of the functional assays undertaken so far and their limitations. A detailed analysis of 38 hERG missense variants identified in Long QT French patients and studied in electrophysiology also underlies the incomplete characterization of the biophysical properties for each variant. These analyses lead to two conclusions: first, the function of many hERG variants has never been looked at and, second, the functional studies done so far are excessively heterogeneous regarding the stimulation protocols, cellular models, experimental temperatures, homozygous and/or the heterozygous condition under study, a context that may lead to conflicting conclusions. The state of the literature emphasizes how necessary and important it is to perform an exhaustive functional characterization of hERG variants and to standardize this effort for meaningful comparison among variants. The review ends with suggestions to create a unique homogeneous protocol that could be shared and adopted among scientists and that would facilitate cardiologists and geneticists in patient counseling and management.

## hERG phenotyping, why?

**FIGURE 1:**
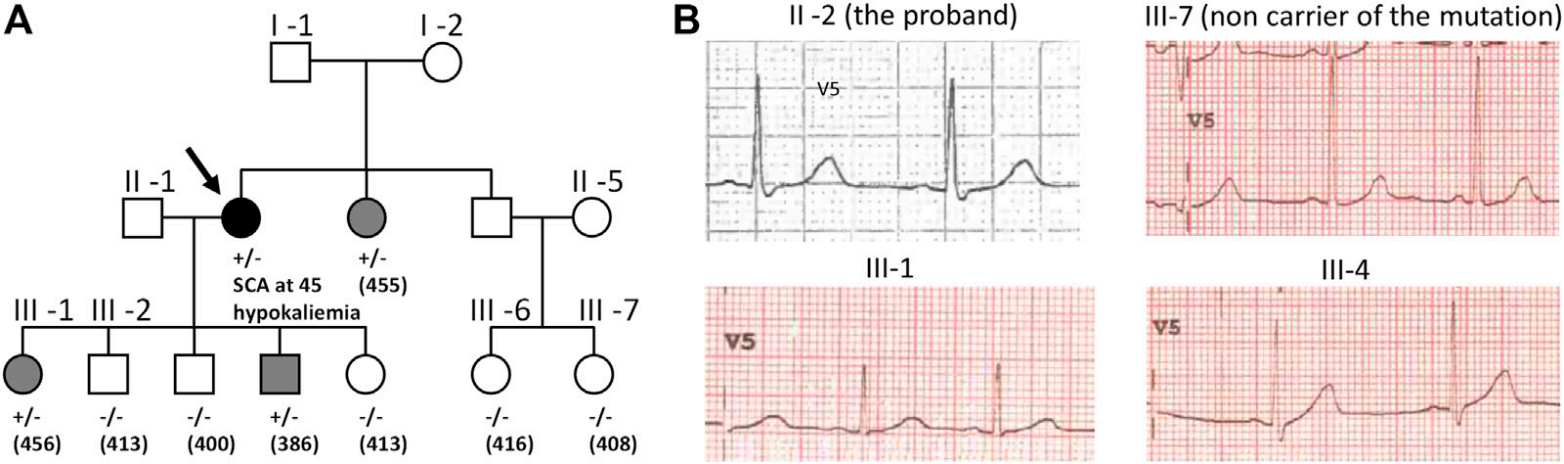
**(A)** Family pedigree harboring the c.2504G>C p.Arg835Pro *KCNH2* mutation that leads to hERG loss of function (Oliveira-Mendes et al., 2021). The proband (arrow) presented a sudden cardiac arrest (SCA) due to ventricular fibrillation at 45 during an episode of hypokalemia requiring an implantable cardiac defibrillator (ICD). Post-Resuscitation ECG was normal. Family members cascade screening detected the *KCNH2* variation in her sister and two children III-1 and III-4; QTc interval values (in ms) are shown between parentheses. Squares depict male subjects; circle, female subject; open symbols, unaffected members; solid black symbol, affected members; grey symbol, mild phenotype. **(B)** ECG tracings in lead V5 of the proband (II-2), a non-carrier niece (III-7), a daughter (III-1) and a son (III-4) who are both carriers of the variant and showing mild LQT2 phenotype (T waves: low amplitude amplitude for III-1 and very slightly bifid for III-4).

The Long QT syndrome (LQTS) is a cardiac disorder characterized by abnormally prolonged ventricular repolarization that results in episodic ventricular tachyarrhythmias sometimes leading to ventricular fibrillation and sudden death in otherwise healthy persons (Charpentier et al., 2010). LQTS is thus a lethal disorder. Symptomatic patients left without therapy had a high mortality rate, 21% within 1 year from the first syncope (Schwartz, 1985). However, mortality rate in properly treated patients has now declined to around 1% over a 10-year period (Schwartz, 2013). Since LQTS is associated with sudden cardiac death, identifying patients at risk is instrumental. Uncovering a pathogenic variant, in one of the genes known to be associated with LQTS (e.g. *KCNH2* gene encoding hERG channel), allows identifying the relatives at risk, in the proband’s family (Alders et al., 1993) (Figure 1A), even when ECGs are not always strongly evocative of the pathology (cf. patient III-1 and patient III-4 in Figure 1B). In absence of robust segregation data, determining the pathogenicity of the variant in the gene sequence of the proband may not be simple. Such variant may be a benign polymorphism that has nothing to do with the patient Long QT syndrome. Hence, *in vitro* functional studies confirming the pathogenicity of the variant will provide critical information.

The American College of Medical Genetics and Genomics (ACMG), the Association for Molecular Pathology (AMP) and the College of American Pathologists (CAP) proposed guidelines that compiles all the parameters that contribute to predict the potential pathogenicity of a given variant, namely the patient phenotype, the segregation data, the conservation of the varying sequence across species, the population data, the *in vitro* functional data, the nature of the amino acids implicated in the sequence variation, the position of the amino-acids, etc. (Richards et al., 2015). The final score classifies a given sequence variation according to five categories: “pathogenic,” “likely pathogenic,” “uncertain significance,” “likely benign,” and “benign.”

The “uncertain significance” (VUS) usually occurs in two situations: when there are conflicting results from the different observables, or when there is insufficient evidence, as is often the case for novel missense variants. Intuitively, it seems reasonable that functional data will be significantly helpful to decrease the number of cases of “uncertain significance.” A theoretical approach proposed by Brnich and collaborators used an algorithm to calculate all combinations of evidence. Using this systematic counting approach, they estimate how functional data allows decreasing the number of VUS. They convincingly illustrate that a majority of VUS could be reclassified with the addition of solid functional data (Brnich et al., 2018). This observation motivates functional studies of the variants identified so far. With the advent of the Next-Generation Sequencing (NGS), the number of variants affecting gene sequences are literally exploding: 99% of the 4.6 million reported missense variations in the Genome Aggregation Database (http://gnomad.broadinstitute.org/) (Lek et al., 2016) are rare (allele frequency <0.005). Interpreting these variants represents a significant roadblock. Only 2% of these variants have a clinical interpretation in ClinVar (Landrum et al., 2014). This observation urges the development of high-throughput and standardized methods for functional characterization of the variants.

**FIGURE 2:**
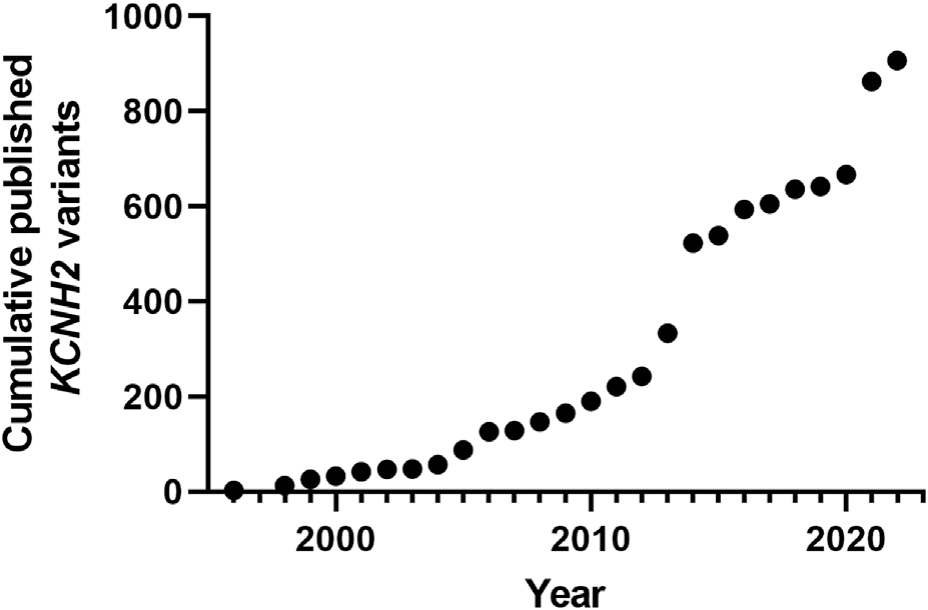
Cumulative published *KCNH2* variants. Years are Pubmed publication years. The two major increases correspond to high-throughput studies, the first focusing on membrane trafficking (Anderson et al., 2014), and the second focusing on ion channel activity with automated patch-clamp experiments (Ng et al., 2020).

At the writing of this review, 2434 *KCNH2* variants are referenced in Clinvar https://www.ncbi.nlm.nih.gov/clinvar/, of which 1832 are associated to LQTS. *KCNH2* gene is associated with type 2 Long QT syndrome, but also with Atrial Fibrillation (Hayashi et al., 2015) and short QT (Brugada et al., 2004). It represents the second most important genetic cause of Long QT syndrome, with an estimated prevalence between 25% and 30% of all cases (Schwartz et al., 2001; Kolder et al., 2015; Kutyifa et al., 2018). Figure 2 illustrates the acceleration of the publication of *KCNH2* variants over the years. As mentioned above, 2434 *KCNH2* variants are referenced in Clinvar, including 1135 missense variants. Since other variants (frameshift, non-sense, splice site) are most often pathogenic, we focused our analyses on missense variants. Among these 1135 missense variants, 962 are classified as VUS, representing approximately 85%.

This review aims at describing the nature of the functional assays undertaken so far and their limits, based on an exhaustive scan of these 1135 missense variants and additional 187 coming from the French network CARDIOGEN, not yet in ClinVar. We also looked at some specific details of the functional assays, in a smaller sample of 112 variants issued from the same French database for type 2 Long QT syndrome patients. This review then comes with new propositions to generate standardized protocols that will help cardiologists and geneticists for patient counseling in face of the increasing number of variants.

## Heterogeneity of the functional assays

As mentioned above, functional data being very helpful to reclassify the VUS, a functional evaluation of hundreds of variants is necessary. Also, the methods of acquisition of these functional data have to be scrupulously standardized to faithfully evaluate the pathogenicity of any variant. In this review, our aim was to establish how far we are from an extensive and standardized evaluation of hERG variants.

**FIGURE 3:**
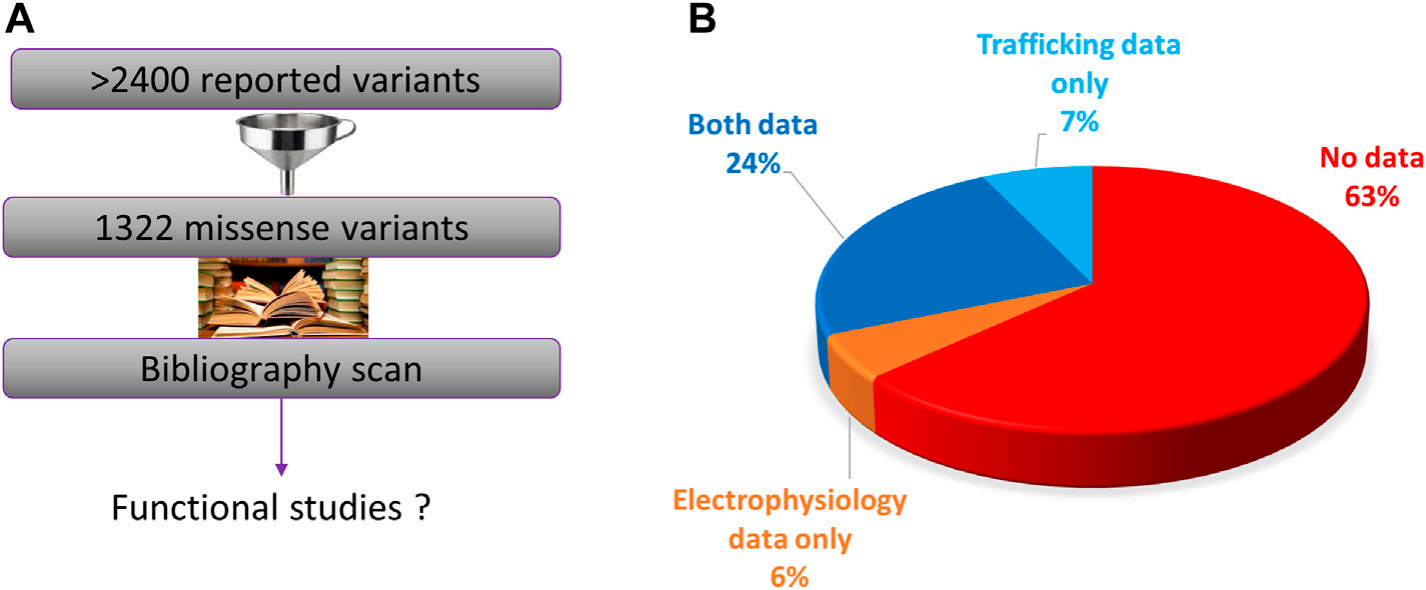
**(A)**. Diagram presenting the principle of the present literature analysis of all the hERG missense variants. **(B)**. Pie chart presenting the percentage of the variants that were not studied at all, studied in electrophysiology only, trafficking studies only, or both (from analysis of Supplemental Table S1).

We performed an exhaustive scan of the literature on the 1322 missense variants (Figure 3A). We can distinguish two kind of functional assays: i) trafficking assays which are only estimating eventual alteration of hERG channel membrane targeting, without giving any keys on channel activity once at the membrane; these assays presented the advantage of between compatible with high-throughput characterization, early on (Anderson et al., 2014). ii) Electrophysiological assays, giving precise and global information on the ion channel activity, whatever the mechanistic nature of the alteration, trafficking and/or gating. We observed that functional assays were performed for only 37% (484/1322) of the variants. In 24% of cases, both trafficking and electrical activity of the variant were evaluated (311/1322) (Figure 3B). In 6% of cases, only electrophysiological experiments were performed, with no parallel trafficking study (74/1322).

Regarding functional studies, it is important to note that patch-clamp is the gold standard for assessing mutations affecting hERG functionality but to date, not all studies have extensively characterized all biophysical aspects. To understand this point, one should know that hERG channels present two gates (one activation gate and one inactivation gate) with specific voltage-dependence and kinetics. In the simplest case, maximal current is evaluated when both activation and inactivation gates are open. If this maximal current is lower in the variant as compared to WT hERG channel, there is a loss of function (Delisle et al., 2005). But despite preserved maximal currents, a potential loss of function of the channel activity may express as an alteration of the voltage-dependence of at least one of the gates: for example, a shift of the activation curve to positive potentials, necessitating greater depolarization to activate the channel, such as for the N470D mutation (Lin et al., 2010). It can also express as a change in channel opening or closing kinetics. For example, hERG N33T, R56Q, G903R, P1075L are associated with faster deactivation kinetics (closing of the activation gate) without any modification of the maximal current amplitude (Ng et al., 2020).

Thus, it is possible that a mutation alters a single parameter among all known biophysical parameters, namely the maximal current amplitude, the half-activation potential, the slope of the activation curve, the half-inactivation potential, the slope of the inactivation curve, activation and deactivation kinetics, inactivation and recovery from inactivation kinetics, and also the ion selectivity. This indicates how critical it is to study systematically all channel parameters of a given variant.

**FIGURE 4:**
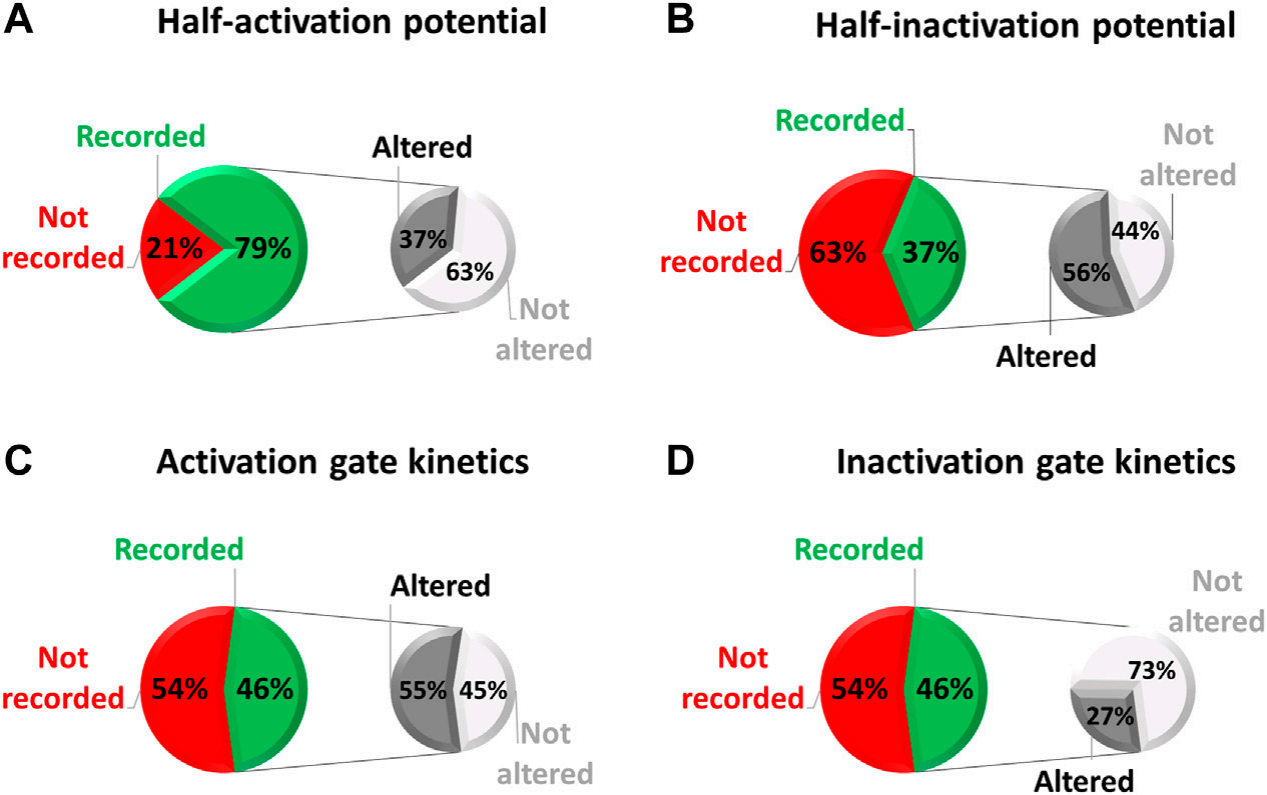
Pie chart presenting, among the missense variants referenced in the French network CARDIOGEN (*n* = 112) and studied in electrophysiology (*n* = 38), the percentage of which half-activation potential **(A)**, half-inactivation potential **(B)**, activation gate kinetics **(C)**, and inactivation gate kinetics **(D)** were studied, and when studied, the percentage of variants showing an alteration of the given parameter (from analysis of Supplemental Table S2).

On a sample of 38 variants that were studied in electrophysiology, we examined how exhaustive all these parameters were characterized. These 38 variants were studied in electrophysiology among a total of 112 missense variants (i.e., 34%) collected in the national database of long QT patients constituted by a network of French Centers of Reference on cardiac arrhythmias (including the ones from Nantes University Hospital and the “Assistance Publique-Hôpitaux de Paris”). These Centers of Reference belong to the French network CARDIOGEN, which has developed a common database extensively reporting clinical and genetic information from all identified French cases. In the case of these 38 missense variants we are far from an extensive study of hERG biophysical parameters (red/green pie charts of Figure 4), despite the evidence that indeed all of the biophysical parameters can be affected individually (monochrome pie charts).

## Experimental temperatures used for functional studies

A literature scan of electrophysiological studies of the 1135 missense variants also illustrates major variations in the temperature applied during two distinct steps of the evaluation of channel variants pathogenicity: 1) during hERG channel expression by the cultured cells, after introduction of the DNA/RNA encoding the channel and 2) during the electrophysiological characterization of the variants.

Temperature at which the cells are cultured during hERG channel expression mainly depends on the expression model used, the two main models being *Xenopus laevis* oocytes, which require incubation at low temperature (12°C–18°C) and mammalian cells, typically incubated at 37°C. Noteworthy, incubation at room temperature rescues the impaired trafficking of many hERG channel variants, as observed by hERG protein glycosylation which is an indicator of correct cell trafficking (Anderson et al., 2014). Functionally, incubation at room temperature may recover channel current amplitudes to values at least similar to WT values (Paulussen et al., 2002). With this knowledge in hand, it seems clear that, *Xenopus laevis* oocytes as the expression model, were not always able to make a logical link between the variant properties and Long QT syndrome.

For example, expression of the LQTS variant R534C, in *X. laevis* oocytes, surprisingly leads to a shift in the activation curve to negative value, predicting thus a shortening in the action potential by a computer model (Nakajima, 1999). In contrast, in mammalian CHO cells, kept at 37°C, R534C is associated with a major decrease in maximal current amplitude that is due to reduced trafficking (Oliveira-Mendes et al., 2021). To reconcile these data, it was shown in HEK293 cells that this reduced trafficking is rescued by incubating the cells at 27°C during the expression time course (Anderson et al., 2006). Since *X. laevis* oocytes are maintained at 12°C–18°C after cRNA injection, it is likely that this temperature favors membrane trafficking of the variant and hence masked the trafficking defect in the aforementioned study (Nakajima, 1999). Thus, the *X. laevis* oocyte model, which was used in 16% of the electrophysiology studies (cf. Figure 6 below) is clearly not the most relevant model to study the pathogenicity of hERG variants and should be avoided in the future.

**FIGURE 5:**
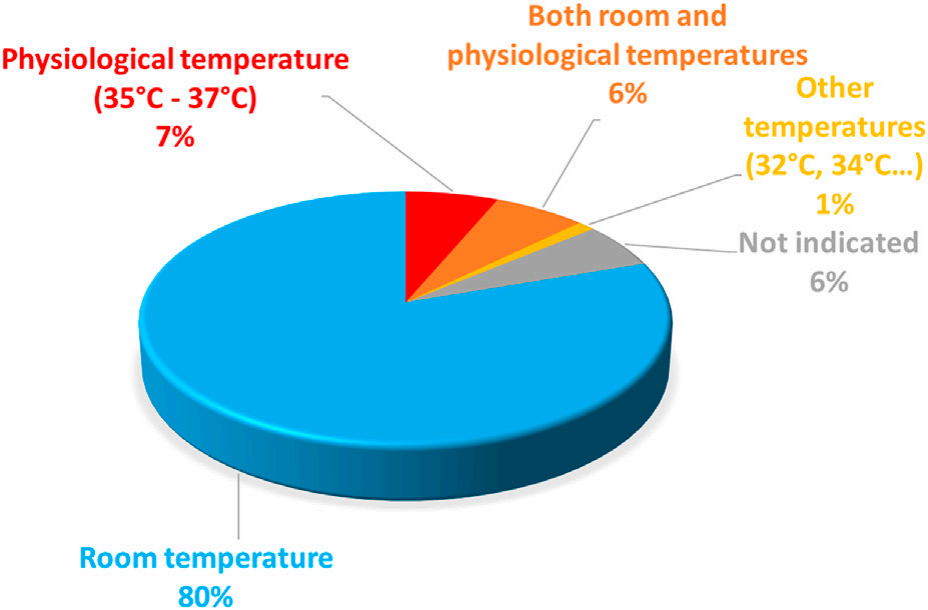
Pie chart presenting the percentage of hERG missense variants (among all the published missense variants), studied at room temperature, at physiological temperature, at both temperatures, or particular temperatures (32°C or 34°C). This chart includes all missense variants studied in patch-clamp experiment performed in mammalian cells (from analysis of Supplemental Table S1).

Regarding the electrophysiological characterization of the variants, they are most frequently done at room temperature (Figure 5). For *Xenopus* oocytes, 37°C is largely above the physiological temperature and hardly tolerable by this cell type (Bienz and Gurdon, 1982). For mammalian cells, room temperature is paradoxically preferred for a technical reason: the success of the experiments (e.g., seal stability) is much higher at room temperature than at 37°C, as illustrated in a study using automated patch-clamp (Ranjan et al., 2019), which shows a success rate of 15% as compared to 80% at room temperature. However, hERG channel biophysical properties, and in particular kinetics such as activation and inactivation kinetics are highly temperature-dependent (Vandenberg et al., 2006). To properly report the biophysical properties of a given hERG variant, it would be wise to study it at 37°C. Alternatively, it would be interesting to keep studying the variant at room temperature and develop a method that would allow extrapolating the evolution of the channel properties with rising temperatures from room temperature to physiological temperature.

## Use of various cell models

**FIGURE 6:**
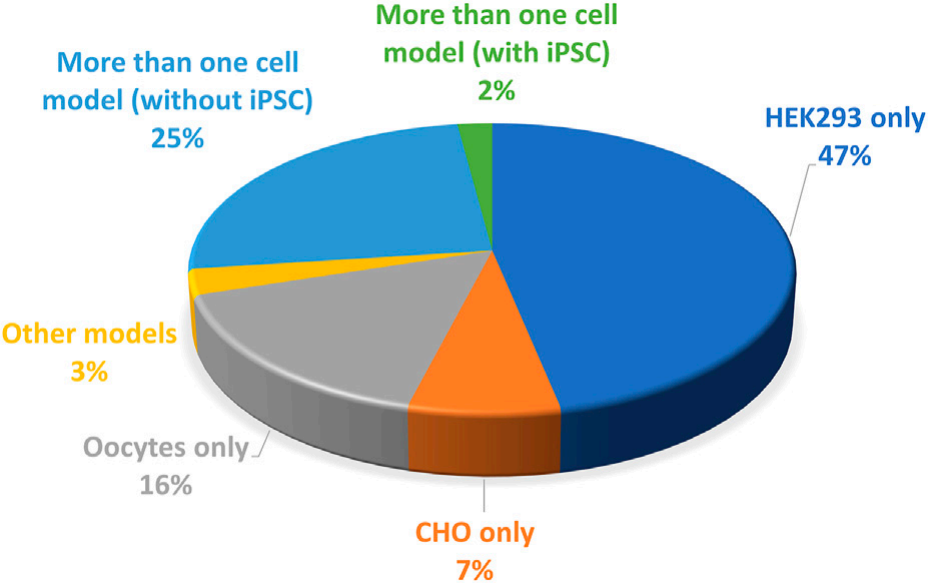
Pie chart presenting the percentage of the cell models used to study the *KCNH2* variants (from analysis of Supplemental Table S1).

Another source of variability is the cell model of study. Three categories of models are used for hERG variants phenotyping (Figure 6):

(i) A non-mammalian model, *X. laevis* oocytes which generate large currents, in the µA range, but have to be maintained at non-physiological temperature which may rescue some mutant impaired trafficking, as developed above. The validity of this model has already been discussed.
(ii) Mammalian cell lines that are easy to maintain and animal-free (van der Velden et al., 2022). Two cell lines are mainly used (CHO, HEK-293). They are cultured at physiological temperature, which represents a clear advantage over *X. laevis* oocytes. The presence of endogenous currents, in these models, but also in *X. laevis* oocytes, may interfere with measurements when studying mutants which generate currents of low amplitudes. To that respect CHO cells are a good model since they expressed minimal voltage-gated currents, when compared to other cells such as HEK-293 cells (Yu and Kerchner, 1998). On the down side, CHO cells proliferate twice faster as HEK-293 cells, potentially leading to much lower currents 48 h after transfection because transfected DNA would be split between more cells (Abaandou et al., 2021). This has to be considered, especially in automated patch-clamp experiments in which one cannot select efficiently transfected cells with a GFP-like reporter gene, as it is routinely done in conventional patch-clamp. Without reporter gene and with high rates of division such as in CHO cells, amplitude may then be too low in many cells for precise characterization of the biophysical parameters.
(iii) Induced pluripotent stem cells developed by Shinya Yamanaka (Takahashi and Yamanaka, 2006) allow the generation of human cardiomyocytes, iPS-CMs (Jouni et al., 2015) that constitute a very relevant model since their genotype is much closer to the patient’s than transfected cells such as CHO or HEK-293 cells. These latter cell lines may be especially limited if the mutant phenotype depends on the presence of a specific auxiliary protein that is not expressed in the cell line, but would be present in native cardiomyocyte. For instance, a study on iPS-derived cardiomyocytes suggests the physiological relevance of the splice variant hERG 1b, which is shorter than the original isolate hERG 1a. hERG 1b, that lacks the N-terminal PAS domain, efficiently associates with hERG 1a, and modifies the generated IKr current (Jones et al., 2014). It remains possible that hERG 1b modifies to some extent the functional effect of at least some variants. For instance, in iPSC-derived cardiomyocytes, the H70R variant of the PAS domain leads to an approximately 50% decrease in current amplitude and an acceleration in deactivation (Feng et al., 2021). At the molecular level, the mutation induces a decrease in hERG 1a membrane trafficking but logically, there is a normal trafficking of hERG 1b lacking the PAS domain and thus the H70R mutation. Such an imbalance between hERG 1b (normal) and hERG 1a (decreased) may theoretically have an impact on the current characteristics.

Are the results obtained with iPS-CMs very different than those obtained in a simpler model and do they justify the use of the more complex, time consuming and costly iPS-derived cardiomyocytes? In *X. laevis* oocytes, the aforementioned mutation (H70R) led to similar deactivation alteration, but had a lower impact (around 25% decrease in homozygous condition) on current amplitude (Chen et al., 1999). Given that *X. laevis* oocytes are not the most relevant model for trafficking-deficient mutants, as mentioned above, the H70R mutation should be extensively studied in patch-clamp, using a simple mammalian model (HEK-293, CHO). So far H70R has only been studied by Western blots from HEK-293 cells, but this technique showed opposing results: one study suggests that H70R hERG is trafficking-deficient (Anderson et al., 2014), whereas another study suggests the opposite (Harley et al., 2012). Hence, it is critical to test whether or not the activities of a given hERG variant evaluated in transfected mammalian cells line and iPS-CMs hERG are very different. Table 1 compares major modifications induced by a variant across various models. More differences can be observed in the *X. laevis* oocytes model as compared to the iPS-CMs model, probably because of the temperature of incubation that rescues the mutation-induced impaired trafficking of the channel, thereby masking or underestimating the effect of the mutation.

**TABLE 1:** Major modifications induced by a variant across various models.

	*Xenopus* oocytes	CHO, HEK-293, COS-7	iPS-CMs	References
R56Q		2-3x acceleration in deactivation	2-3x acceleration in deactivation	Liu and Trudeau (2015)
H70R	25% decrease in current		50% decrease in current	Chen et al. (1999), Feng et al. (2021)
	2x acceleration in deactivation		2x acceleration in deactivation	
R534C	No decrease in current in heterozygous condition		50% decrease in current	Nakajima (1999), Mesquita et al. (2019)
A561P		70% decrease in current in heterozygous condition	50% decrease in current	Bellocq et al. (2004), Jouni et al. (2015)
		10-mV shift of the activation curve	No shift of the activation curve	
G601S	40% decrease in current	85% decrease in current		Furutani et al. (1999)

Adding a level of complexity, it is possible that the current level is not always a quantitative marker of the pathology, even in iPS-CMs. One study identified two modifier genes in a family presenting a highly variable severity of the LQT among its members (carrying the R752W mutant), but similar hERG current levels in their corresponding iPS-CMs (Chai et al., 2018). Another study was also able to mimic, at the action potential level, the patient-dependent severity of the LQTS due to the L552S mutation in hERG despite similar current levels in asymptomatic vs. symptomatic patients iPS-CMs (Shah et al., 2020). Nevertheless, more studies are needed to firmly conclude on the added value of the more complex, time-consuming and costly iPS-derived cardiomyocytes in most of the variants. Noteworthy, patient-specific iPS-CMs is not a resource that all investigators have access to. Another option is to use commercially available iPS-CMs cell lines which can be infected with hERG variants to study their biophysical characteristics in a cardiac cell type (Liu and Trudeau, 2015).

The use of iPS-CMs may be more critical in other pathologies such as the Brugada syndrome in which the cell environment/patient’s genome is more influent. For instance, iPS-CMs generated from six Brugada patients with different genetic backgrounds, revealed the same I_Na_ current abnormality in the six cell lines, independently of the presence (2 Brugada patients) or absence (4 Brugada patients) of a mutation in the *SCN5A* gene, coding for the sodium channel Na_V_1.5 (Al Sayed et al., 2021). This suggests that iPS-CMs are critical to study the mechanisms implicated in complex polygenic pathologies such as the Brugada syndrome (Barc et al., 2022).

The generation of iPS-CMs may in the future allow identifying the impact of a variant in a hERG channel regulator, such as KCNE2 which has been proposed as a potential accessory subunit for hERG, but this remains to be confirmed (Eldstrom and Fedida, 2011). If KCNE2 is confirmed as an auxiliary subunit, co-transfection of hERG with this subunit in a mammalian cell line may add more precision to this kind of hERG phenotyping by allowing the system to be more similar to the patient cardiomyocyte. On the other hand, any hypothetical accessory subunit has to be carefully validated first. Moreover, relative expression of hERG vs*.* the auxiliary subunit has to be carefully calibrated since non-physiological overexpression of this subunit can rescue a loss of function mutation on the channel subunit, and underestimate the effect of a mutant, a story quite similar to the use of room temperature in *X. laevis* oocytes experiments described above (Liu et al., 2016).

At last, some articles use neonatal cardiomyocytes, as in a study which described similar effect of the G601S and N470D mutations on the current characteristics as in HEK-293 cells, for instance on the activation curve and deactivation kinetics (Lin et al., 2010).

## Is it possible to easily mimic heterozygosity in high-throughput systems?

Importantly, most LQT2 mutation carriers are heterozygous. For instance, less than 1% of probands registered in the French database from the CARDIOGEN network are homozygous for a mutation. In order to mimic as much as possible, the condition of the patient, it is useful 1) to determine if there is an equal expression of both alleles, which seems to be the case (Shah et al., 2020), and 2) to be able to reproduce in the model of study this equal expression of both alleles.

In conventional patch-clamp study, equal expression of both alleles is achieved by transfecting cells with the equal amount of the two DNAs (Ficker et al., 2000; Chevalier, 2001; Saenen et al., 2007; Hayashi et al., 2009; McBride et al., 2013). Mimicking such heterozygosity is not always performed, some studies only comparing the WT and mutant condition, without testing the WT + mutant co-expression (22% of all variants studied in electrophysiology are only studied in the homozygote condition, cf. Supplemental Table S1). Classical transient transfections, such as FuGENE, Lipofectamine based transfections, are not very efficient in term of the percentage of transfected cells, requiring the co-expression of a reporter such as GFP to select the transgene expressing cells. In a high-throughput system, it is not possible to detect and select a transfected cell, leading to two major options: i) electroporation, which is much more efficient than chemical transfection in term of the percentage of transfected cells, has been successfully used for KCNQ1 high-throughput phenotyping (Vanoye et al., 2018). ii) A cell line stably expressing the WT or mutant channel can overcome the need of transient transfection and was used for hERG high-throughput phenotyping (Ng et al., 2020). Yet, one first limit is that production of stable cell lines is time consuming (several steps of antibiotic selections). Second, such strategy allows the integration of a unique plasmid in cells, representing a limit as compared to transient transfection, which allows transfection of 2 (or more) plasmids, as mentioned above. Thus, the heterozygote situation can be mimicked only by the use of a plasmid with two expression cassettes, provided that expressions of the two cDNA are equivalent. This requirement does not seem to be respected in several studies that make use of bicistronic plasmids containing an Internal Ribosome Entry Site (IRES), in which expression of the first cassette is at least five time greater than expression in the second cassette (Mizuguchi et al., 2000). Of note, a study suggests it may be possible to reduce the difference by mutating the IRES (Al-Allaf et al., 2019). If this strategy is successfully applied for equal expression of hERG cDNA in the two cassettes, it would be a worthwhile recommendation for the study of hERG variants.

## Artifactual heterogeneity of the recordings

It is critical to identify the potential artifacts leading to misevaluation of the effects of a given variant on the current characteristics and hence on the channel biophysical properties. A study using a mathematical model elegantly suggested that variability in hERG-channel-independent parameters (such as leak current, series resistance, etc.) is responsible for a major variability in the observed biophysical parameters (Lei et al., 2020). We also mathematically modeled the variability of the biophysical parameters of a voltage-dependent potassium channel, studied in conventional and high-throughput patch-clamp channels (Montnach et al., 2021). From the results we obtained, we proposed to reduce such variability, by limiting maximal current amplitude and series resistance. Beyond such maximal values, insufficient voltage-clamp generates variability in the voltage-dependence of activation. Our model also pinpoints that a phenomenon observed in several K_v_ channels and named “delayed repolarization” is in fact an artifactual property due to insufficient voltage-clamp. This strongly suggests that, in the case of transient transfection, the protocol has to be finely tuned to prevent currents larger than 10 nA (Montnach et al., 2021).

In the same vein, in order to limit the artifactual heterogeneity of the recordings, it is also critical to design the most adapted voltage simulation protocol specific to a given channel, since voltage-dependence and kinetics drastically vary from one channel type to another. For instance, a steady-state activation protocol in which the depolarizing pulse are too short in time may lead to erroneous effect of a given variant, such as the negative shift in the activation curve in the case of the R176W variant (Oliveira-Mendes et al., 2021).

Up to now, a majority of studies focused on a limited number of biophysical parameters among the following: the maximal current amplitude, the half-activation potential, the slope of the activation curve, the half-inactivation potential, the slope of the inactivation curve, activation and deactivation kinetics, inactivation and recovery from inactivation kinetics, and also the ion selectivity. Standardized and complete protocols, allowing a fast and quasi-exhaustive characterization of hERG channel activity, will prove more insightful than those studies. Such a protocol, used in (Oliveira-Mendes et al., 2021) can also be very short (35-s). This shortness is another criterion of robustness, giving little chance for cell characteristics such as the seal quality, series resistance, current amplitude, to vary during the course of the experiment. Another interest of such fast protocol is that it can be used to track concomitant variations of several parameters (e.g., amplitude, half-activation potential, deactivation). We used a simplified version of this complex protocol, to be able to follow the concomitant variations of several biophysical parameters caused by the variation in the membrane level of the membrane phospholipid PIP_2_. Such a great deal of data allowed us to add many numerical constraints on a kinetic model and to demonstrate that PIP_2_ stabilizes hERG channel open state (Rodriguez et al., 2010).

Another similar strategy to maximize the amount of information gathered with minimal time required is to use an unconventional protocol (such as a sinusoidal voltage-clamp protocol for conventional patch-clamp or a staircase protocol compatible with high-throughput system) generating enough information on the current characteristics, to use it to constrain a kinetic model that will be accurate enough to describe the properties of the activation and inactivation gates. Impressively, the protocol is able to predict the current obtained by the classical protocols (Beattie et al., 2018; Lei et al., 2019b). A major interest is that such approach may also be used to predict channel behavior at 37°C from experiments done at room temperature, which are much more successful than experiments at 37°C, as exposed above (Lei et al., 2019a). It remains to be determined if this unconventional protocol (sinusoidal; staircase), tested on the WT channel, is robust enough for the prediction of the biophysical behavior of channel variants. The 35-s protocol mentioned above, which does not need a kinetic model, has already been proven to be robust to study the electrophysiological activity of channel variants (Oliveira-Mendes et al., 2021).

## *In silico* phenotyping

The guidelines proposed by the ACMG, AMP and CAP, mentioned above include criteria issued from *In Silico* pathogenicity prediction softwares, which are evaluating the evolutionary conservation of an amino acid or nucleotide, the location and context within the protein sequence, and the biochemical consequence of the amino acid substitution (Richards et al., 2015). Such tools are far from being 100% reliable (Riuró et al., 2015), but new effort have been engaged to improve the robustness of the prediction using deep learning (Qi et al., 2021).

The Cryo-EM structure of hERG channel in the open state represents an instrumental template on which molecular variants can be introduced to test their propensity to affect channel structure and hence function. One major limit is that the actual sequence lacks non-negligible intracellular regions of the channel (Wang and MacKinnon, 2017). It is though worth noting that hERG channel characteristics are not completely disturbed by the deletions operated in the cytoplasmic domain (Zhang et al., 2020). But it is clear that using such structure will be more predictive of a variant phenotype when the whole structure will be solved, in the different states (open, closed and inactivated) and in presence of potential hERG channel partners.

## Conclusion

Our analysis of the large body of published work on hERG variants highlighted a lack of standardized analysis for hERG variants functional studies, which prevents an accurate comparison of the variants pathogenicity. In the future, a gold standard approach should use a single cell model, that must be a mammalian cell model (e.g., CHO or HEK293) and not *X. laevis* oocytes, and should mimic the heterozygote situation. iPS-derived human cardiomyocytes should be used as much as possible to further validate/invalidate such a simple heterologous expression system. In addition, electrophysiological recordings should be performed at physiological temperature, but it is still rarely the case, most probably due to the impact of temperature on seal quality, a problem that will be difficult to address. Also, standardized cell transfection protocols should be used to limit current amplitude and consequently incorrect voltage-clamp. Finally, standardized and optimized voltage-clamp protocol should be used to limit variability in the results. Recent initiatives using high-throughput electrophysiology are naturally going in that direction. This new tool represents a way to re-visit the body of variants in a much more standardized way. In complement to this standardization in the functional characterization, it is also important to standardize variant interpretation, as suggested by the Clinical Genome Resource Sequence Variant Interpretation Working Group and a laboratory working on *KCNH2* variant high-throughput phenotyping (Brnich et al., 2020; Jiang et al., 2022).

At last, development of *In Silico* methods will logically bring in parallel a standardized approach to predict the functional impact of a given variant.

## References

[B1] Abaandou L. Quan D. Shiloach J. (2021). Affecting HEK293 cell growth and production performance by modifying the expression of specific genes. Cells 10, 1667. 10.3390/cells10071667 34359846 PMC8304725

[B2] Al Sayed Z. R. Jouni M. Gourraud J. Belbachir N. Barc J. Girardeau A. (2021). A consistent arrhythmogenic trait in Brugada syndrome cellular phenotype. Clin. Transl. Med. 11, e413. 10.1002/ctm2.413 34185406 PMC8181201

[B3] Al-Allaf F. A. Abduljaleel Z. Athar M. Taher M. M. Khan W. Mehmet H. (2019). Modifying inter-cistronic sequence significantly enhances IRES dependent second gene expression in bicistronic vector: Construction of optimised cassette for gene therapy of familial hypercholesterolemia. Non-coding RNA Res. 4, 1–14. 10.1016/j.ncrna.2018.11.005 PMC6404380 30891532

[B4] Alders M. Bikker H. Christiaans I. (1993). “Long QT syndrome,” in GeneReviews®. Editors Adam M. P. Everman D. B. Mirzaa G. M. Pagon R. A. Wallace S. E. Bean L. J. (Seattle, Seattle (WA: University of Washington). 20301308

[B5] Anderson C. L. Delisle B. P. Anson B. D. Kilby J. A. Will M. L. Tester D. J. (2006). Most LQT2 mutations reduce Kv11.1 (hERG) current by a class 2 (Trafficking-Deficient) mechanism. Circulation 113, 365–373. 10.1161/CIRCULATIONAHA.105.570200 16432067

[B6] Anderson C. L. Kuzmicki C. E. Childs R. R. Hintz C. J. Delisle B. P. January C. T. (2014). Large-scale mutational analysis of Kv11.1 reveals molecular insights into type 2 long QT syndrome. Nat. Commun. 5, 5535. 10.1038/ncomms6535 25417810 PMC4243539

[B7] Barc J. Tadros R. Glinge C. Chiang D. Y. Jouni M. Simonet F. (2022). Genome-wide association analyses identify new Brugada syndrome risk loci and highlight a new mechanism of sodium channel regulation in disease susceptibility. Nat. Genet. 54, 232–239. 10.1038/s41588-021-01007-6 35210625 PMC9376964

[B8] Beattie K. A. Hill A. P. Bardenet R. Cui Y. Vandenberg J. I. Gavaghan D. J. (2018). Sinusoidal voltage protocols for rapid characterisation of ion channel kinetics: Sinusoidal protocols to capture ion channel kinetics. J. Physiol. 596, 1813–1828. 10.1113/JP275733 29573276 PMC5978315

[B9] Bellocq C. Wilders R. Schott J.-J. Louérat-Oriou B. Boisseau P. Le Marec H. (2004). A common antitussive drug, clobutinol, precipitates the long QT syndrome 2. Mol. Pharmacol. 66, 1093–1102. 10.1124/mol.104.001065 15280442

[B10] Bienz M. Gurdon J. B. (1982). The heat-shock response in xenopus oocytes is controlled at the translational level. Cell 29, 811–819. 10.1016/0092-8674(82)90443-3 6891290

[B11] Brnich S. E. Abou Tayoun A. N. Couch F. J. Cutting G. R. Greenblatt M. S. Heinen C. D. (2020). Recommendations for application of the functional evidence PS3/BS3 criterion using the ACMG/AMP sequence variant interpretation framework. Genome Med. 12, 3. 10.1186/s13073-019-0690-2 PMC6938631 31892348

[B12] Brnich S. E. Rivera Muñoz E. A. Berg J. S. (2018). Quantifying the potential of functional evidence to reclassify variants of uncertain significance in the categorical and Bayesian interpretation frameworks. Hum. Mutat. 39, 1531–1541. 10.1002/humu.23609 30095857 PMC6548460

[B13] Brugada R. Hong K. Dumaine R. Cordeiro J. Gaita F. Borggrefe M. (2004). Sudden death associated with short-QT syndrome linked to mutations in HERG. Circulation 109, 30–35. 10.1161/01.CIR.0000109482.92774.3A 14676148

[B14] Chai S. Wan X. Ramirez-Navarro A. Tesar P. J. Kaufman E. S. Ficker E. (2018). Physiological genomics identifies genetic modifiers of long QT syndrome type 2 severity. J. Clin. Investigation 128, 1043–1056. 10.1172/JCI94996 PMC5824853 29431731

[B15] Charpentier F. Mérot J. Loussouarn G. Baró I. (2010). Delayed rectifier K+ currents and cardiac repolarization. J. Mol. Cell. Cardiol. 48, 37–44. 10.1016/j.yjmcc.2009.08.005 19683534

[B16] Chen J. Zou A. Splawski I. Keating M. T. Sanguinetti M. C. (1999). Long QT syndrome-associated mutations in the per-arnt-sim (PAS) domain of HERG potassium channels accelerate channel deactivation. J. Biol. Chem. 274, 10113–10118. 10.1074/jbc.274.15.10113 10187793

[B17] Chevalier P. Rodriguez C. Bontemps L. Miquel M. Kirkorian G. Rousson R. (2001). Non-invasive testing of acquired long QT syndrome Evidence for multiple arrhythmogenic substrates. Cardiovasc. Res. 50, 386–398. 10.1016/S0008-6363(01)00263-2 11334843

[B18] Delisle B. P. Slind J. K. Kilby J. A. Anderson C. L. Anson B. D. Balijepalli R. C. (2005). Intragenic suppression of trafficking-defective KCNH2 channels associated with long QT syndrome. Mol. Pharmacol. 68, 233–240. 10.1124/mol.105.012914 15851652

[B19] Eldstrom J. Fedida D. (2011). The voltage-gated channel accessory protein KCNE2: Multiple ion channel partners, multiple ways to long QT syndrome. Expert Rev. Mol. Med. 13, e38. 10.1017/S1462399411002092 22166675

[B20] Feng L. Zhang J. Lee C. Kim G. Liu F. Petersen A. J. (2021). Long QT syndrome *KCNH2* variant induces hERG1a/1b subunit imbalance in patient-specific induced pluripotent stem cell–derived cardiomyocytes. Circ Arrhythmia Electrophysiol. 14, e009343. 10.1161/CIRCEP.120.009343 PMC8058932 33729832

[B21] Ficker E. Thomas D. Viswanathan P. C. Dennis A. T. Priori S. G. Napolitano C. (2000). Novel characteristics of a misprocessed mutant HERG channel linked to hereditary long QT syndrome. Am. J. Physiol. Heart Circ. Physiol. 279, H1748–H1756. 10.1152/ajpheart.2000.279.4.H1748 11009462

[B22] Furutani M. Trudeau M. C. Hagiwara N. Seki A. Gong Q. Zhou Z. (1999). Novel mechanism associated with an inherited cardiac arrhythmia: Defective protein trafficking by the mutant HERG (G601S) potassium channel. Circulation 99, 2290–2294. 10.1161/01.CIR.99.17.2290 10226095

[B23] Harley C. A. Jesus C. S. H. Carvalho R. Brito R. M. M. Morais-Cabral J. H. (2012). Changes in channel trafficking and protein stability caused by LQT2 mutations in the PAS domain of the HERG channel. PLoS ONE 7, e32654. 10.1371/journal.pone.0032654 22396785 PMC3292575

[B24] Hayashi K. Fujino N. Uchiyama K. Ino H. Sakata K. Konno T. (2009). Long QT syndrome and associated gene mutation carriers in Japanese children: Results from ECG screening examinations. Clin. Sci. 117, 415–424. 10.1042/CS20080528 19371231

[B25] Hayashi K. Konno T. Tada H. Tani S. Liu L. Fujino N. (2015). Functional characterization of rare variants implicated in susceptibility to lone atrial fibrillation. Circ. Arrhythm. Electrophysiol. 8, 1095–1104. 10.1161/CIRCEP.114.002519 26129877

[B26] Jiang C. Richardson E. Farr J. Hill A. P. Ullah R. Kroncke B. M. (2022). A calibrated functional patch-clamp assay to enhance clinical variant interpretation in KCNH2-related long QT syndrome. Am. J. Hum. Genet. 109, 1199–1207. 10.1016/j.ajhg.2022.05.002 35688147 PMC9300752

[B27] Jones D. K. Liu F. Vaidyanathan R. Eckhardt L. L. Trudeau M. C. Robertson G. A. (2014). hERG 1b is critical for human cardiac repolarization. Proc. Natl. Acad. Sci. U.S.A. 111, 18073–18077. 10.1073/pnas.1414945111 25453103 PMC4273358

[B28] Jouni M. Si Tayeb K. Es Salah Lamoureux Z. Latypova X. Champon B. Caillaud A. (2015). Toward personalized medicine: Using cardiomyocytes differentiated from urine derived pluripotent stem cells to recapitulate electrophysiological characteristics of type 2 long QT syndrome. JAHA 4, e002159. 10.1161/JAHA.115.002159 26330336 PMC4599503

[B29] Kolder I. C. R. M. Tanck M. W. T. Postema P. G. Barc J. Sinner M. F. Zumhagen S. (2015). Analysis for genetic modifiers of disease severity in patients with long-QT syndrome type 2. Circ. Cardiovasc Genet. 8, 447–456. 10.1161/CIRCGENETICS.114.000785 25737393 PMC4770255

[B30] Kutyifa V. Daimee U. A. McNitt S. Polonsky B. Lowenstein C. Cutter K. (2018). Clinical aspects of the three major genetic forms of long QT syndrome (LQT1, LQT2, LQT3). Ann. Noninvasive Electrocardiol. 23, e12537. 10.1111/anec.12537 29504689 PMC5980697

[B31] Landrum M. J. Lee J. M. Riley G. R. Jang W. Rubinstein W. S. Church D. M. (2014). ClinVar: Public archive of relationships among sequence variation and human phenotype. Nucl. Acids Res. 42, D980–D985. 10.1093/nar/gkt1113 24234437 PMC3965032

[B32] Lei C. L. Clerx M. Beattie K. A. Melgari D. Hancox J. C. Gavaghan D. J. (2019a). Rapid characterization of hERG channel kinetics II: Temperature dependence. Biophysical J. 117, 2455–2470. 10.1016/j.bpj.2019.07.030 PMC6990152 31451180

[B33] Lei C. L. Clerx M. Gavaghan D. J. Polonchuk L. Mirams G. R. Wang K. (2019b). Rapid characterization of hERG channel kinetics I: Using an automated high-throughput system. Biophysical J. 117, 2438–2454. 10.1016/j.bpj.2019.07.029 PMC6990155 31447109

[B34] Lei C. L. Clerx M. Whittaker D. G. Gavaghan D. J. de Boer T. P. Mirams G. R. (2020). Accounting for variability in ion current recordings using a mathematical model of artefacts in voltage-clamp experiments. Phil. Trans. R. Soc. A 378, 20190348. 10.1098/rsta.2019.0348 32448060 PMC7287334

[B35] Lek M. Karczewski K. J. Minikel E. V. Samocha K. E. Banks E. Fennell T. (2016). Analysis of protein-coding genetic variation in 60,706 humans. Nature 536, 285–291. 10.1038/nature19057 27535533 PMC5018207

[B36] Lin E. C. Holzem K. M. Anson B. D. Moungey B. M. Balijepalli S. Y. Tester D. J. (2010). Properties of WT and mutant hERG K ^+^ channels expressed in neonatal mouse cardiomyocytes. Am. J. Physiology-Heart Circulatory Physiology 298, H1842–H1849. 10.1152/ajpheart.01236.2009 PMC2886621 20363883

[B37] Liu L. Tian J. Lu C. Chen X. Fu Y. Xu B. (2016). Electrophysiological characteristics of the LQT2 syndrome mutation KCNH2-g572S and regulation by accessory protein KCNE2. Front. Physiol. 7, 650. 10.3389/fphys.2016.00650 28082916 PMC5187237

[B38] Liu Q. Trudeau M. C. (2015). Eag domains regulate LQT mutant hERG channels in human induced pluripotent stem cell-derived cardiomyocytes. PLoS ONE 10, e0123951. 10.1371/journal.pone.0123951 25923442 PMC4414485

[B39] McBride C. M. Smith A. M. Smith J. L. Reloj A. R. Velasco E. J. Powell J. (2013). Mechanistic basis for type 2 long QT syndrome caused by KCNH2 mutations that disrupt conserved arginine residues in the voltage sensor. J. Membr. Biol. 246, 355–364. 10.1007/s00232-013-9539-6 23546015 PMC3706098

[B40] Mesquita F. C. P. Arantes P. C. Kasai-Brunswick T. H. Araujo D. S. Gubert F. Monnerat G. (2019). R534C mutation in hERG causes a trafficking defect in iPSC-derived cardiomyocytes from patients with type 2 long QT syndrome. Sci. Rep. 9, 19203. 10.1038/s41598-019-55837-w 31844156 PMC6915575

[B41] Mizuguchi H. Xu Z. Ishii-Watabe A. Uchida E. Hayakawa T. (2000). IRES-dependent second gene expression is significantly lower than cap-dependent first gene expression in a bicistronic vector. Mol. Ther. 1, 376–382. 10.1006/mthe.2000.0050 10933956

[B42] Montnach J. Lorenzini M. Lesage A. Simon I. Nicolas S. Moreau E. (2021). Computer modeling of whole-cell voltage-clamp analyses to delineate guidelines for good practice of manual and automated patch-clamp. Sci. Rep. 11, 3282. 10.1038/s41598-021-82077-8 33558601 PMC7870888

[B43] Nakajima T. Furukawa T. Hirano Y. Tanaka T. Sakurada H. Takahashi T. (1999). Voltage-shift of the current activation in HERG S4 mutation (R534C) in LQT2. Cardiovasc. Res. 44, 283–293. 10.1016/S0008-6363(99)00195-9 10690305

[B44] Ng C.-A. Perry M. D. Liang W. Smith N. J. Foo B. Shrier A. (2020). High-throughput phenotyping of heteromeric human ether-à-go-go-related gene potassium channel variants can discriminate pathogenic from rare benign variants. Heart rhythm. 17, 492–500. 10.1016/j.hrthm.2019.09.020 31557540

[B45] Oliveira Mendes B. Feliciangeli S. Ménard M. Chatelain F. Alameh M. Montnach J. (2021). A standardised hERG phenotyping pipeline to evaluate *KCNH2* genetic variant pathogenicity. Clin. Transl. Med. 11, e609. 10.1002/ctm2.609 34841674 PMC8609418

[B46] Paulussen A. Raes A. Matthijs G. Snyders D. J. Cohen N. Aerssens J. (2002). A novel mutation (T65P) in the PAS domain of the human potassium channelchannel HERG results in the long QT syndrome by trafficking deficiency. J. Biol. Chem. 277, 48610–48616. 10.1074/jbc.M206569200 12354768

[B47] Qi H. Zhang H. Zhao Y. Chen C. Long J. J. Chung W. K. (2021). MVP predicts the pathogenicity of missense variants by deep learning. Nat. Commun. 12, 510. 10.1038/s41467-020-20847-0 33479230 PMC7820281

[B48] Ranjan R. Logette E. Marani M. Herzog M. Tâche V. Scantamburlo E. (2019). A kinetic map of the homomeric voltage-gated potassium channel (Kv) family. Front. Cell. Neurosci. 13, 358. 10.3389/fncel.2019.00358 31481875 PMC6710402

[B49] Richards S. Aziz N. Bale S. Bick D. Das S. Gastier-Foster J. (2015). Standards and guidelines for the interpretation of sequence variants: A joint consensus recommendation of the American College of medical genetics and genomics and the association for molecular pathology. Genet. Med. 17, 405–424. 10.1038/gim.2015.30 25741868 PMC4544753

[B50] Riuró H. Campuzano O. Berne P. Arbelo E. Iglesias A. Pérez-Serra A. (2015). Genetic analysis, *in silico* prediction, and family segregation in long QT syndrome. Eur. J. Hum. Genet. 23, 79–85. 10.1038/ejhg.2014.54 24667783 PMC4266740

[B51] Rodriguez N. Amarouch M. Y. Montnach J. Piron J. Labro A. J. Charpentier F. (2010). Phosphatidylinositol-4,5-Bisphosphate (PIP2) stabilizes the open pore conformation of the Kv11.1 (hERG) channel. Biophysical J. 99, 1110–1118. 10.1016/j.bpj.2010.06.013 PMC2920645 20712994

[B52] Saenen J. B. Paulussen A. D. C. Jongbloed R. J. Marcelis C. L. Gilissen R. A. H. J. Aerssens J. (2007). A single hERG mutation underlying a spectrum of acquired and congenital long QT syndrome phenotypes. J. Mol. Cell. Cardiol. 43, 63–72. 10.1016/j.yjmcc.2007.04.012 17531263

[B53] Schwartz P. J. (1985). Idiopathic long QT syndrome: Progress and questions. Am. Heart J. 109, 399–411. 10.1016/0002-8703(85)90626-X 3966369

[B54] Schwartz P. J. Priori S. G. Spazzolini C. Moss A. J. Vincent G. M. Napolitano C. (2001). Genotype-phenotype correlation in the long-QT syndrome: Gene-specific triggers for life-threatening arrhythmias. Circulation 103, 89–95. 10.1161/01.cir.103.1.89 11136691

[B55] Schwartz P. (2013). Practical issues in the management of the long QT syndrome: Focus on diagnosis and therapy. Swiss Med. Wkly. 143, w13843. 10.4414/smw.2013.13843 24089242

[B56] Shah D. Prajapati C. Penttinen K. Cherian R. M. Koivumäki J. T. Alexanova A. (2020). hiPSC-derived cardiomyocyte model of LQT2 syndrome derived from asymptomatic and symptomatic mutation carriers reproduces clinical differences in aggregates but not in single cells. Cells 9, 1153. 10.3390/cells9051153 32392813 PMC7290503

[B57] Takahashi K. Yamanaka S. (2006). Induction of pluripotent stem cells from mouse embryonic and adult fibroblast cultures by defined factors. Cell 126, 663–676. 10.1016/j.cell.2006.07.024 16904174

[B58] van der Velden J. Asselbergs F. W. Bakkers J. Batkai S. Bertrand L. Bezzina C. R. (2022). Animal models and animal-free innovations for cardiovascular research: Current status and routes to be explored. Consensus document of the ESC working Group on myocardial function and the ESC working Group on cellular biology of the heart. Cardiovasc. Res. cvab370 118, 3016–3051. 10.1093/cvr/cvab370 PMC9732557 34999816

[B59] Vandenberg J. I. Varghese A. Lu Y. Bursill J. A. Mahaut-Smith M. P. Huang C. L.-H. (2006). Temperature dependence of human *ether-à-go-go-* related gene K ^+^ currents. Am. J. Physiology-Cell Physiology 291, C165–C175. 10.1152/ajpcell.00596.2005 16452156

[B60] Vanoye C. G. Desai R. R. Fabre K. L. Gallagher S. L. Potet F. DeKeyser J.-M. (2018). High-throughput functional evaluation of *KCNQ1* decrypts variants of unknown significance. Circ Genomic Precis. Med. 11, e002345. 10.1161/CIRCGEN.118.002345 PMC6309341 30571187

[B61] Wang W. MacKinnon R. (2017). Cryo-EM structure of the open human ether-à-go-go -related K + channel hERG. Cell 169, 422–430. 10.1016/j.cell.2017.03.048 28431243 PMC5484391

[B62] Yu S. P. Kerchner G. A. (1998). Endogenous voltage-gated potassium channels in human embryonic kidney (HEK293) cells. J. Neurosci. Res. 52, 612–617. 10.1002/(SICI)1097-4547(19980601)52:5<612::AID-JNR13>3.0.CO;2-3 9632317

[B63] Zhang Y. Dempsey C. E. Hancox J. C. (2020). Electrophysiological characterization of the modified hERG _T_ potassium channel used to obtain the first cryo‐EM hERG structure. Physiol. Rep. 8, e14568. 10.14814/phy2.14568 33091232 PMC7580876

